# High-Resolution Genetics Identifies the Lipid Transfer Protein Sec14p as Target for Antifungal Ergolines

**DOI:** 10.1371/journal.pgen.1006374

**Published:** 2016-11-17

**Authors:** Ireos Filipuzzi, Simona Cotesta, Francesca Perruccio, Britta Knapp, Yue Fu, Christian Studer, Verena Pries, Ralph Riedl, Stephen B. Helliwell, Katarina T. Petrovic, N. Rao Movva, Dominique Sanglard, Jianshi Tao, Dominic Hoepfner

**Affiliations:** 1 Novartis Institutes for BioMedical Research, Novartis Campus, Basel, Switzerland; 2 Genomics Institute of the Novartis Research Foundation, San Diego, California, United States of America; 3 Institute of Microbiology, University of Lausanne and University Hospital Center, Lausanne, Switzerland; Duke University School of Medicine, UNITED STATES

## Abstract

Invasive infections by fungal pathogens cause more deaths than malaria worldwide. We found the ergoline compound NGx04 in an antifungal screen, with selectivity over mammalian cells. High-resolution chemogenomics identified the lipid transfer protein Sec14p as the target of NGx04 and compound-resistant mutations in Sec14p define compound-target interactions in the substrate binding pocket of the protein. Beyond its essential lipid transfer function in a variety of pathogenic fungi, Sec14p is also involved in secretion of virulence determinants essential for the pathogenicity of fungi such as *Cryptococcus neoformans*, making Sec14p an attractive antifungal target. Consistent with this dual function, we demonstrate that NGx04 inhibits the growth of two clinical isolates of *C*. *neoformans* and that NGx04-related compounds have equal and even higher potency against *C*. *neoformans*. Furthermore NGx04 analogues showed fungicidal activity against a fluconazole resistant *C*. *neoformans* strain. In summary, we present genetic evidence that NGx04 inhibits fungal Sec14p and initial data supporting NGx04 as a novel antifungal starting point.

## Author Summary

Emerging resistance to antibiotics led to an inglorious revival of infectious diseases. Furthermore, in the past 30 years, only one novel anti-fungal target has been discovered which was used to develop therapies against. Therefore pathogen-selective targets and knowledge about possible resistance determinants are of utmost importance to successfully develop new medicines. Here we describe the identification of anti-fungal ergolines, targeting the lipid transfer protein Sec14p, and inhibiting the growth of two clinical isolates of the pathogenic fungus *Cryptococcus neoformans*. Both, compound and target represent attractive points for further investigations: Sec14p as it differs significantly from the human homolog and as it has been implicated in fungal viability and pathogenicity, and, ergolines as they are used in the clinic against a variety of diseases demonstrating both efficacy and safety.

## Introduction

Severe invasive fungal infections (IFIs) are life-threatening and associated with high mortality [1]. *Candida albicans* (mortality: 20–50%) [2], *Cryptococcus neoformans* (mortality: 20–70%) [3] and *Aspergillus fumigatus* (mortality: 50–90%) [4] represent the major human fungal pathogens that have devastating effects on mostly, but not exclusively, immune compromised individuals such as HIV patients, premature infants, diabetics, chemotherapy patients, organ transplant recipients and aging people [1, 5]. Currently marketed drugs suffer from limited efficacy and bioavailability, narrow species spectrum and high toxicity [6, 7] and the development of novel drugs against fungal-selective protein targets is challenging due to the high evolutionary conservation between fungi and man. This results in a limited number of druggable nodes and thus restricted chemical matter [8]. Only one new antifungal drug class to treat systemic infections has been approved in the US in the last three decades, the β-glucan synthase inhibitors echinocandins (reviewed in [9]). Efficacy of echinocandins however is limited, as they suffer from poor bioavailability [10]. New fungal-selective targets are thus needed to screen for novel chemotypes to fight these infections [6, 8].

The virulence of *C*. *neoformans* is largely dependent on its secretion machinery, as many of the molecules required to exert its virulence are extracellular [11, 12]. Secreted factors include the major polysaccharide building block of the capsule, enzymes involved in the assembly, maintenance and integrity of the cell wall as well as other proteins [13–17]. So far three Sec proteins, Sec4p, Sec6p and Sec14p, have been implicated in secretion of factors essential to virulence of *C*. *neoformans* [12]. Among these three Sec proteins, the phosphatidylinositol and phosphatidylcholine transfer protein Sec14p is most likely druggable [18], whereas Sec4p, a Rab family member, or Sec6p, an anchoring protein, represent by far more challenging target classes like GTPases and protein-protein interactions, respectively. Sec14p has been shown to be essential not only for virulence, but also for growth [11], a dual role which makes this enzyme an attractive drug target. Here we show the characterization of a fungal Sec14p inhibitor by using a suite of genetic assays to de-convolute the mode of action of the screening hit, NGx04, prioritized due to an established track record of ergolines in medicine [19–25]. Chemogenomic profiling identified hypersensitivity and resistance of deletion strains related to the action of Sec14p. Saturating mutagenesis of the SEC14 gene identified four key residues that reduced NGx04 potency. All identified residues localized into the lipid-binding cavity of the Sec14 protein suggesting that NGx04 is an active-site inhibitor and competition with the Sec14p lipid substrates supported this hypothesis. Resistance-residue guided *in silico* docking of NGx04 into the lipid-binding cavity outlined critical sites for compound-protein interaction most of which are conserved in the protein sequence of the pathogen *C*. *neoformans* but not in the human homolog. Testing NGx04 against two clinical isolates of *C*. *neoformans* confirmed antifungal action and lack of pronounced cytotoxicity in human cells. Interestingly, the most potent NGx04 derivative was found to exert fungicidal activity against a strain resistant to fluconazole, a commonly used drug against cryptococcosis.

In summary, we present evidence that NGx04 inhibits fungal Sec14p and initial data supporting NGx04 as a novel antifungal starting point.

## Results

### Haploinsufficiency profiling (HIP) and homozygous profiling (HOP) of NGx04

**Fig 1:**
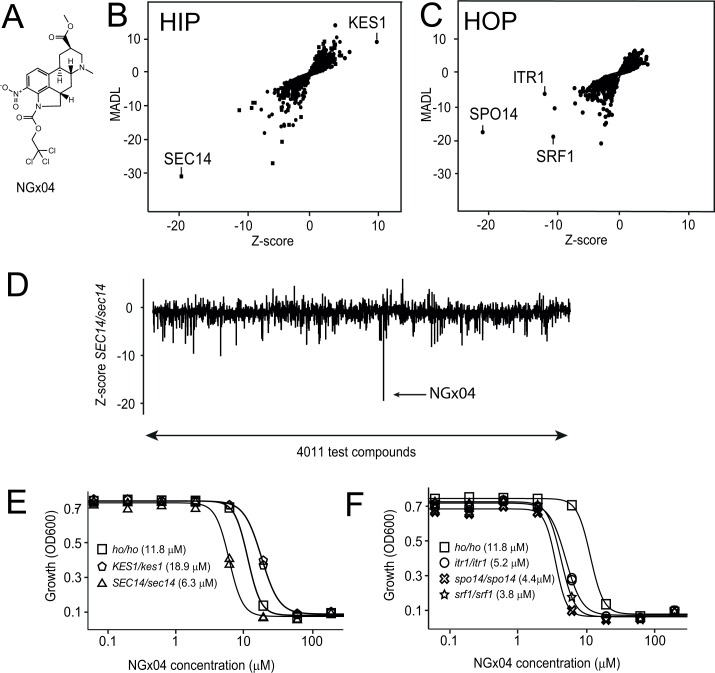
Chemogenomic profiling. **Structure, haploinsufficiency profiling (HIP) and homozygous profiling (HOP) of NGx04.** A) The structure of NGx04 is shown. B) HIP and C) HOP were performed around IC_30_ (8 μM) in duplicates and the average of the profiles is shown. The MADL score is given on the y-axis and indicates how sensitive a particular deletion strain is to a given compound, compared to its no-drug control. The Z-score is given on the x-axis and is a statistical measure of how frequently a particular deletion strain is affected within the so far profiled compounds [26]. Circles depict non-essential deletions, whereas squares essential deletions. Hyper-sensitive deletions are found bottom left and hyper-resistant deletions top right of the panels. D) Hypersensitivity of a heterozygous *SEC14/sec14* strain across 4011 diverse compounds (x-axis) is displayed as Z-score (y-axis). NGx04 (arrow) was found to exert the strongest effect. Validation of the hit candidates from HIP E), HOP F) was performed by single strain growth inhibition. The indicated strains were grown for 24 h in the presence of increasing concentrations of NGx04 and growth measured by turbidity (OD_600_). Duplicate values were determined and used for logistic regression. The calculated IC_50_ values are displayed.

A high-throughput screen of the Novartis compound archive against *S*. *cerevisiae* was performed to identify novel antifungal compounds [26, 27]. The ergot-related compound NGx04 (Fig 1A) was among the validated hits scoring an IC_50_ < 20 μM. Due to the long history of ergolines in medicine [19–25] this scaffold was selected for target identification by chemogenomic profiling [26]. Haploinsufficiency profiling (HIP) and homozygous profiling (HOP) are gene-dosage-dependent methods that assess the effect(s) of compounds against potential targets encoded by the *S*. *cerevisiae* genome [28]. HIP indicates pathways directly affected by the compound, whereas HOP, with both gene copies deleted, indicates synthetic lethality and identifies compensating pathways to those directly affected by the compound. Testing NGx04 at sub-lethal doses (8 μM) in two independent biological replicates and correlating the profiles as previously published [26] identified the heterozygous deletion of *SEC14* to result in significantly reduced fitness compared to the rest of the genome-wide heterozygous deletion collection (HIP) (Fig 1B). In contrast, heterozygous deletion of *KES1*, which encodes a protein that negatively regulates Sec14p-dependent secretion [29, 30], resulted in increased fitness (Fig 1B). Hypersensitivity of the heterozygous *SEC14* strain was found to be unique when analyzing the scores of these two strains in our database capturing profiles of > 4000 diverse compounds (Fig 1D). Chemical-synthetic interactions of NGx04 (HOP) were identified with homozygous deletions of the genes encoding the regulatory subunit (*SRF1*) and the catalytic subunit (*SPO14*) of Phospholipase D, a previously identified substrate of Sec14p [31, 32], and *ITR1* (Fig 1C), the major myo-inositol transporter in *S*. *cerevisiae* [33]. Hypersensitivity of *SEC14/sec14*, *srf1/srf1*, *spo14/spo14 or itr1/itr1* and hyposensitivity of *KES1/kes1* against NGx04 was confirmed by titrating NGx04 across the deletion strains and comparing inhibition to the control stain *ho/ho* (Fig 1E HIP and Fig 1F HOP). Taken together, this data suggests that NGx04 interferes with cellular viability at the level of the phosphatidylinositol and phosphatidylcholine transfer protein Sec14p.

### Functional variomics screen for NGx04-resistant mutants

**Fig 2:**
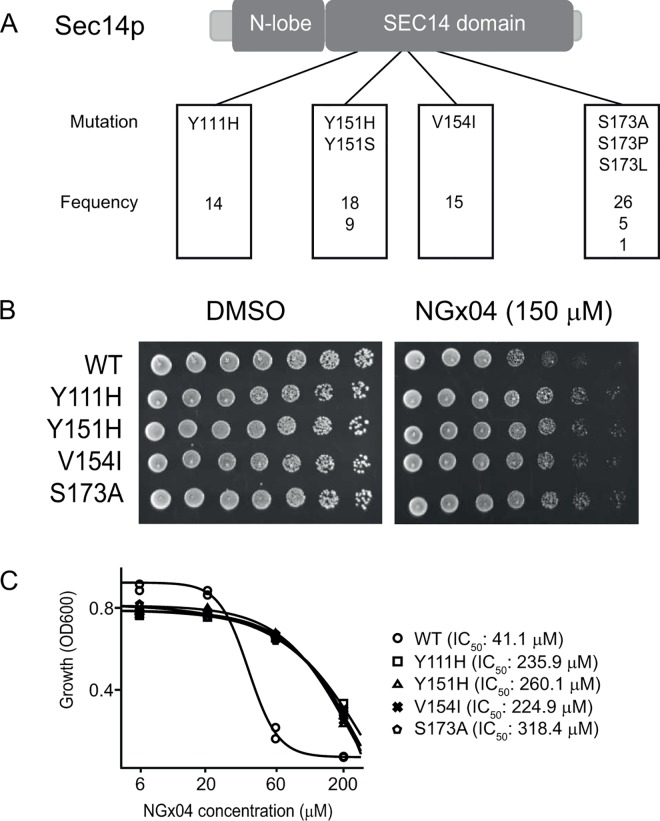
Resistance mapping. **Functional variomics screen for NGx04-resistant mutants.** A) The SEC14 ORFs of 88 NGx04-resistant colonies were amplified by PCR and sequenced. Four mutations mapping to the lipid-binding part of Sec14p were found. The identified amino acid changes with their respective frequencies are given. B) The plasmids coding for various SEC14 proteins were re-transformed into a fresh wild type yeast strain and serial dilutions of the cultures spotted on either 150 μM of NGx04 or DMSO control, incubated for 3 days at 30°C and scored for growth. C) The strains were grown for 24 h in the presence of increasing concentrations of NGx04 and growth measured by turbidity (OD_600_). Duplicate values were determined and the 18 h time point was used for logistic regression. The calculated IC_50_ values displayed.

To verify our findings from the chemogenomic profiling studies, a saturation mutagenesis screening approach [34] was used and supported Sec14p as the target of NGx04. A pool containing approximately 10^5^ plasmid-based, mutagenized Sec14p-variants [34] was plated onto solid growth media supplemented with 150 μM NGx04, a concentration that prevented colony formation of wild-type cells. Eighty-eight surviving colonies were isolated, plasmid DNA encoding the Sec14 proteins isolated and sequenced. This approach yielded mutations in four distinct amino acids of Sec14p: Y111H (identified 14 times), Y151H/S (identified 27 times), V154I (identified 15 times) and S173A/P/L (identified 32 times) (Fig 2A). To confirm that the mutations were the resistance-defining factors, the plasmids containing the *SEC14* mutants were retransformed into wild type yeast. A plasmid containing the wild type copy of *SEC14* was used as control to exclude resistance due to *SEC14* gene copy-number effects. Spotting for single colony formation on lethal doses of NGx04 (Fig 2B) as well as growth inhibition titrations (Fig 2C) revealed that each of the four identified mutations was sufficient to confer resistance to NGx04. As the cells contained also the endogenous wild type *SEC14* allele this data revealed that the drug resistance was dominant and provided further support that Sec14p is the growth inhibitory target of NGx04.

### Phosphatidylinositol (PI) and phosphatidylcholine (PC) competition

**Fig 3:**
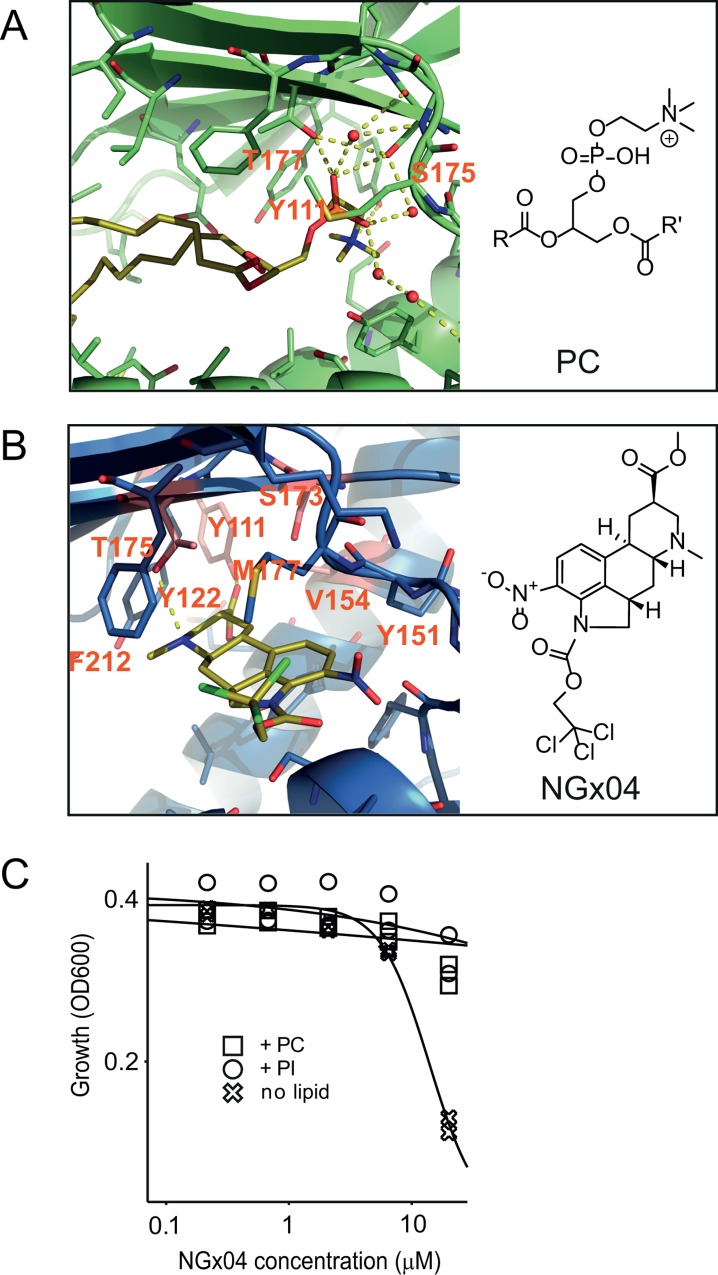
Active site antagonism. **Phosphatidylinositol (PI) and phosphatidylcholine (PC) competition.** A) The crystal structure of Phosphatidylcholine (PC) bound to the yeast Sfh1protein (PDB ID: 3B7Q), (where R is oleoyl and R’ is palmitol). PC hydrophobic tail forms van der Waals interactions with a cluster of hydrophobic residue, whereas the phosphate moiety is interacting with the side-chain hydroxyl groups of residues S175 and T177, and the ethanolamine nitrogen contacts the phenolic oxygen of Y113. B) The proposed docking pose of NGx04 in the putative binding site of the yeast protein structure is shown (PDB ID:1AUA). In the suggested binding mode, the ester moiety of the ligand forms a hydrogen bond with Y111 while the ergoline amine forms H-bonds with the T175. The ergoline moiety is also forming van der Waals interactions with the following residues: Y122, Y151, S173, M177 and F212. C) Wild type yeast was grown in presence of increasing concentrations of NGx04, supplemented with either 200 μM PI, 200 μM PC or no phospholipid, incubated at 30°C and growth followed over time by measuring turbidity (OD_600_). Duplicate values were determined and the time points at 18 h were used for logistic regression.

Crystal structures of Sec14p and Sec14p-like proteins from various species including *S*. *cerevisisae* (PDB ID: 1AUA) and human (PDB ID: 1OLM) have been solved. Although the lipid-binding pocket of the enzyme is well characterized [35–37] Sec14p has not been crystalized when bound to phosphatidylethanolamine (PtdEtn) substrate. The crystal structure of the S. cerevisisae Sfh1protein (PDB ID: 3B7Q), sharing 62.3% identity with Sec14p, shows that phosphatidylcoline (PC) hydrophobic tail forms van der Waals interactions with a cluster of hydrophobic residues, whereas the phosphate moiety is interacting with the side-chain hydroxyl groups of residues S175 and T177, and the nitrogen contacts the phenolic oxygen of Y113 (Fig 3A). Since the NGx04 resistance-conferring residues were located in the equivalent catalytic site of Sec14p (Fig 3A) (i.e. S173, corresponding to S175, and T175 to T177 in Sfh1, two residues involved in H-bonds with PC) we reasoned that NGx04 would bind in the lipid-binding pocket of the protein.

To test this hypothesis, the *S*. *cerevisisae* x-ray structure of Sec14p was used to dock the NGx04 ligand into the lipid-binding cavity (Fig 3B). In the resulting docking model, the ester moiety of the ligand formed an H-bond with Y111 while the ergoline amine formed an H-bond with T175. The ergoline moiety also made van der Waals interactions with the residues Y122, Y151, S173, M177 and F212. The calculated docking model was in agreement with the experimental data from the mutagenesis study, as three of the four identified resistance conferring mutations were directly involved in ligand interactions (Y111, Y151 and S173) and the identified V154 residue, although not directly involved in ligand interactions, was only 4.5 Å away from NGx04. The model outlines that a larger residue at this position like Isoleucine would be deleterious for ligand binding. As our docking experiment was biased towards the lipid-binding cavity we aimed at further supporting evidence for NGx04 binding in this protein pocket. To do so we tested if NGx04 would compete for phospholipid-binding in the Sec14p cavity. Wild type *S*. *cerevisiae* cells were grown in presence of increasing doses of NGx04 in medium containing an excess of either phosphatidylinositol (PI) or phosphatidylcholine (PC) (200 μM). Both lipids, PI and PC, were found to alleviate NGx04-mediated growth inhibition, supporting a competitive mode of inhibition at the lipid-binding cavity of Sec14 (Fig 3C).

### Mammalian cell proliferation and cytotoxicity of NGx04

**Fig 4:**
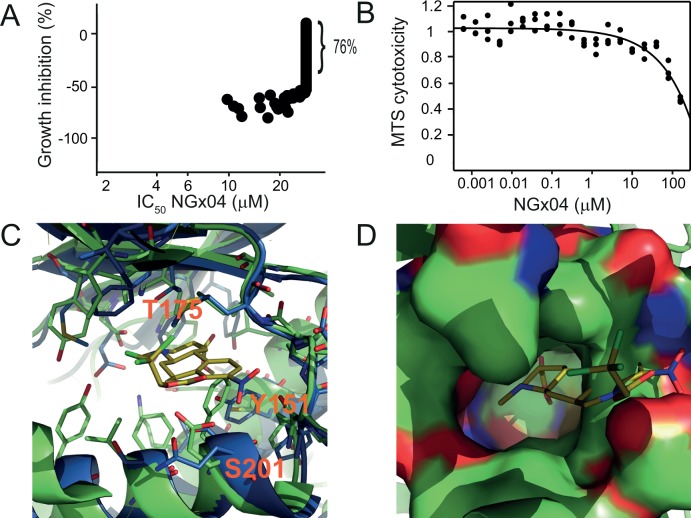
Fungal selectivity. **Mammalian cell growth inhibition and cytotoxicity of NGx04.** A) Dose-response curves for NGx04 were generated in growth inhibition assay and IC_50_ values determined in duplicates [39]. IC_50_ values and growth inhibition (Amax) are displayed. 76% of the tested cell lines were not significantly inhibited by NGx04. B) A dose-response curves for NGx04 was generated in a MTS cytotoxicity assay using HepG2 cells in triplicates and a toxicity curve generated by logistic regression. C) Overlay of the crystal structure of the yeast Sec14p (PDB ID: 1AUA, protein structure highlighted in blue) and its human orthologue SEC14L2 (PDB ID: 1OLM, protein structure highlighted in green) reveals that the amino acid residues belonging to the putative binding site are not conserved between the yeast and the human protein. D) Overlay of the docked NGx04 in the yeast Sec14p (PDB ID: 1AUA) and its human orthologue SEC14L2 (PDB ID: 1OLM). The amino acid residues of the yeast protein structure have been hidden whereas the surface of the putative binding site of the human protein structure has been added to highlight the difference in the overall shape between the active site of the two species.

One key requirement of antifungal medicines is selective inhibition of the pathogen over the mammalian host. To explore potential undesired activity of NGx04 against mammalian cells we used the Cancer Cell Line Encyclopedia CCLE, a collection of 512 human cancer cell lines (broadinstitute.org/ccle). Dose-response curves for NGx04 were generated in a proliferation assay and IC_50_ values determined. Although a large number of anti-proliferative compounds were published to be active across the CCLE collection [38, 39] only one fourth of the cell lines was found to be sensitive to NGx04 as defined by Amax <50% and IC_50_ <30 μM. Furthermore the large majority of cells (76%) were not notably affected by NGx04 (Fig 4A). Since HepG2 cells are known to be a suitable tool to profile toxicity of compounds [40], we used this liver-derived line to assess cytotoxicity of NGx04. A dose-response curve was generated and an IC_50_ value of 157.6 μM determined by logistic regression (Fig 4B), again indicating potential selectivity of NGx04 for the fungal protein. Superimposition of the *S*. *cerevisisae* Sec14p crystal structure (PDB ID: 1AUA, blue) and the crystal structure of the human orthologue SEC14L2 (PDB ID: 1OLM, green) (Fig 4C) revealed that although having a similar overall structure fold, the active site of the two enzymes differ considerably. 16 of 21 amino acid residues within a 5 Å radius from any atom of the docked Ngx04 ligand were not conserved between the two proteins. Critical differences in the active site of the human protein are the residues F178, C128, I151 and L186 (corresponding to S201, Y151, T175 and M209 in yeast) which form a smaller and more hydrophobic pocket compared to the yeast protein (Fig 4C). Visualizing the solvent accessible surface of the human Sec14p with NGx04 as a potential ligand revealed that NGx04 is too big to occupy this smaller binding pocket (see clear ligand bumps towards the solvent accessible surface) (Fig 4D). In addition, all four NGx04-interacting amino acids identified by functional variomics are missing in the human enzyme (S1 Fig). In summary, it is plausible that the observed lack of potent activity of NGx04 against human cell lines can be explained by differences in the catalytic pocket and the absence of proposed NGx04 interacting residues in the human protein.

### *C*. *neoformans* growth inhibition by NGx04 scaffolds

**Fig 5:**
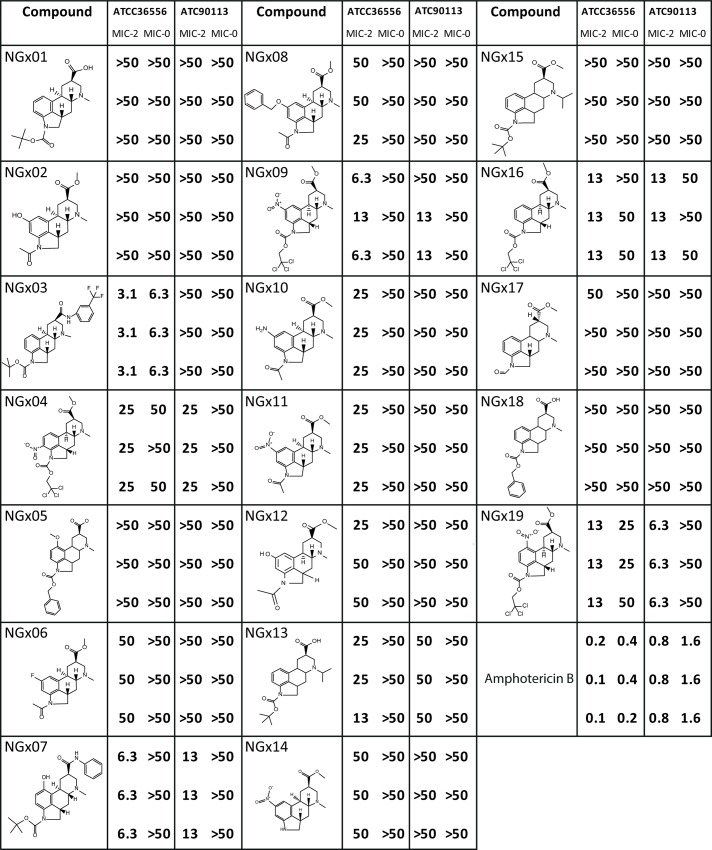
Antifungal testing. ***C*. *neoformans* growth inhibition by NGx04 scaffolds.** NGx04 and close analogues were tested against *C*. *neoformans* ATCC 36556 and ATCC 90113 in a 72 h proliferation assay and MIC-2 (50% inhibition) and MIC-0 (no visible fungal growth) determined. Triplicate values are given in μg/ml. Amphotericin B was included as positive control.

Sec14p is reported to be essential for virulence as well as for growth in *C*. *neoformans* [11, 12]. The *S*. *cerevisiae* and *C*. *neoformans* proteins show 53% similarity by pair-wise alignment with higher conservation of the residues in the lipid-binding pocket (S2 Fig). Furthermore, three of the four sites identified to be critical for NGx04 sensitivity, are conserved (S2 Fig). In combination with the current high unmet medical need related to *Cryptococcus* infections we prioritized testing of NGx04 and 18 related ergoline scaffold analogs against this pathogen. Two clinical isolates of *C*. *neoformans* (ATCC 36556 and ATCC 90113) were plated in triplicates on increasing drug concentrations and MIC-2 (50% inhibition) and MIC-0 (no visible fungal growth) concentrations were scored after 72 hrs. (Fig 5). NGx04 and analogues including NGx03, NGx16, NGx07, NGx09 and NGx19 were found to inhibit *C*. *neoformans* growth in the tested dose range. NGx03 appeared to be the most potent compound against ATCC 36556 with a MIC-2 of 3.13 μg/ml and a MIC-0 of 6.25 μg/ml whereas NGx19 showed a broader spectrum with a MIC-2 of 12.5μg/ml against the first isolate and a MIC-2 of 6.25μg/ml against the second strain.

Fluconazole (FLU) is commonly used to treat cryptococcosis [41]. Repeated exposure or prolonged treatment with azoles, however, can lead to resistance [42–44]. We thus tested the ability of the most potent compounds to inhibit growth of the FLU-resistant *C*. *neoformans* strain BPY22 [45] and found activity for NGx03 (MIC-2 of 16 μg/ml and MIC-0 of 32 μg/ml) and weaker activity for NGx16 and NGx19 (S3 Fig), indicating the potential of ergolines to fight FLU-resistant *C*. *neoformans*. We next assessed the mechanism of inhibition, since fungicidal agents, compared to fungistatic agents [46], are associated with decreased mortality and found NGx03 and NGx16 to exhibit a fungicidal effect on FLU-sensitive and FLU-resistant *C*. *neoformans* strains (S6 Fig). NGx03 was among the most active compound when tested in susceptibility assays and thus corroborates with its fungicidal effect in the tested strains.

Analysis of the structure-activity relationship (SAR) of the tested ergolines (Fig 5) revealed that analogues where the aromatic amino group was substituted by a 2,2,2-trichloroethyl-carboxylate moiety scored anti-fungal activity (NGx03 and NGx07), while the analogues with an unsubstituted, acetylated or formulated aromatic amino group lacked notable activity. Nitro substitution of the aromatic ring appeared to be tolerated in all positions as the analogue without any substituent was found to be equipotent. In summary, antifungal activity against *C*. *neoformans* as predicted by the analysis of identified NGx04 interacting residues could experimentally be confirmed. Our limited SAR experiment identified that building off the aromatic ring was tolerated enabling future medicinal chemistry approaches with the aim to assess and optimize potency, spectrum and other pharmacokinetic parameters of this scaffold.

## Discussion

Severe IFIs are often life-threatening conditions. The clinical anti-fungal arsenal however is very limited and the β-glucan synthase inhibitor echinocandin is the only drug developed over the last three decades that modulates a novel target. In addition, the emergence of resistance against established treatments dramatically emphasizes the importance of identifying novel and selective drug targets. *C*. *neoformans* is the most common cause of fungal meningitis (reviewed in [47], and the invasion of the central nervous system by *C*. *neoformans* is strictly dependent on the secretion of proteins like phospholipase B1 (Plb1) [11], the metalloprotease Mpr1 [48], building blocks of the capsule, and enzymes involved in the assembly, maintenance and integrity of the cell wall as well as other proteins [12] [49]. Protein traffic and secretion in *C*. *neoformans* has been described to occur via more than one pathway involving either Sec4p, Sec6p or Sec14p [12], where the secretion of the key cryptococcal virulence factor Plb1 is strictly Sec14p-dependent [11]. Of the three Sec proteins involved, the phosphatidylinositol and phosphatidylcholine transfer protein Sec14p represents the most promising target, with the first inhibitory scaffold published recently [18]. Sec4p, a Rab family GTPase, is very difficult to target, and several pharmaceutical laboratories failed in the past to identify selective molecules against this target family. Sec6p, an essential subunit of the exocyst complex is a structural protein which could be modulated by interfering with its interactions to binding partners, however, tackling protein-protein interactions is difficult and often yields only very limited success.

Genomic fitness analysis has proven to be a powerful approach for mode of action elucidation of test compounds [26, 28, 50–52]. Whereas haploinsufficiency profiling (HIP) can directly identify the target of a test compound, homozygous profiling (HOP) identifies synthetic lethal and compensatory interactions. Follow up work of a large high-throughput screening campaign by HIP HOP identified NGx04 as a potential Sec14p inhibitor. No chemical probe, interfering with either Sec4p or Sec6p was found among the ca 4,000 acquired compound profiles. The heterozygous *SEC14* deletion showed dramatically reduced fitness at sub-lethal NGx04 doses (HIP), indicating that reducing Sec14p expression represents the main action of NGx04. Interestingly, the heterozygous deletion of Kes1p, a protein which negatively regulates Sec14p-dependent secretion [29, 30], led to increased fitness as deletion of a repressor appeared to activate the pathway. The homozygous deletions of the regulatory subunit (Srf1p) and the catalytic subunit (Spo14p) of PLD were found to be synthetic lethal with inhibiting Sec14p by NGx04. PLD was described to bypass Sec14 mutations [53] by generating a phosphatidic acid pool that is utilized in supporting yeast Golgi secretory function. In addition, deletion of Itr1p, a Myo-inositol sugar transporter, was also found to be synthetic lethal with Sec14p inhibition, probably by depletion of inositol levels in the cell. Thus, our data indicate that NGx04 interferes with Sec14p directly or might modulate a Sec14p-dependent process.

Target identification and validation by resistance cloning has often been successful since mutations in the drug binding pocket of the target can lead to lack of drug binding and thus resistance. Spontaneous or chemically induced, random mutations were historically screened for resistance followed by cloning of the responsible single nucleotide polymorphism (SNP) [54]. An elegant variation of the approach has been published and termed *functional variomics* [34]. The functional variomics tool consists of a (nearly) genome-wide collection of plasmid-based SNPs. Over 90% of the yeast ORFs are represented as pools of 10,000 to 100,000 different SNPs. Plating the functional variomics pool of *SEC14* at inhibitory concentrations of NGx04, led to the identification of four distinct SNPs, isolated multiple times. Plating the genome-wide functional variomics pool, led to the identification of only *SEC14*-related mutations (S4 Fig), supporting that Sec14p is the efficacy target of NGx04. All isolated SEC14 SNPs, Y111H, Y151H/S, V154I and S173A/P/L, located to the lipid-binding pocket of Sec14p, indicating that NGx04 was a substrate-competitive Sec14p inhibitor. Crystal structures of Sec14 proteins from various species, including *S*. *cerevisiae* [55], have been solved and the lipid-binding pocket of the enzyme is well characterized [35–37]. The residues S173 and T175 are involved in the coordination of the phosphate moiety whereas the phenolic oxygen of Y111 binds the nitrogen the substrate PtdEtn. As Y111H and S173A give resistance to NGx04, we postulated that the compound competes for substrate binding which we showed by PI- and PC-mediated suppression of NGx04-induced growth inhibition. The observed dominant resistance by single mutations is a concern when considering NGx04as an anti-fungal lead compound. However, advanced knowledge on privileged sites than can confer resistance is valuable information and we believe that a medicinal chemistry program paralleled by the functional variomics approach will allow the further optimization of NGx04-derived compounds with favorable resistance profiles.

As a precursor for *in vivo* toxicology studies we assessed the potential of NGx04 to inhibit proliferation of human cells by determining IC_50_ values across the Cancer Cell Line Encyclopedia. Most of the tested lines were not found to be significantly sensitive and, in addition, a HepG2-based cytotoxicity assay confirmed this finding. Genetic complementation of an *S*.*cerevisiae* SEC14 homozygous deletion by human SEC14 failed in our hands (S5 Fig), probably due to evolutionary divergence. Although the lipid transfer function of Sec14 proteins has been conserved during evolution, structural differences are found between the *S*. *cerevisiae* and human proteins [56]. The two proteins show a similar overall protein fold, however, specific differences are found in the lipid-binding pocket where amino acids critical for NGx04 binding are not conserved between the human and the fungal proteins [57]. These differences affect the overall shape of the putative binding site, forming a smaller binding pocket in the human active site. A *C*. *neoformans* Sec14p structure is needed to further exploit species-specific features and guide medicinal chemistry. Aligning *S*.*cerevisiae* SEC14 to *C*. *neoformans* SEC14-1 and SEC14-2 shows that most residues involved in NGx04 binding as well as responsible for mutation-based resistance are conserved (Y111, Y122, Y151, S173 and T175) (S2 Fig), suggest that NGx04 binds to the two fungal proteins in a very similar way.

*In vitro* activity against two clinical isolates of *C*. *neoformans* was assessed. NGx04 scored anti-cryptococcal activity and more potent derivatives were identified. Substitutions of the aromatic amino group of the scaffold were found to be critical for its anti-fungal activity, whereas other residues had less effect. Two features seem to determine potency of the compound class, the chlorinated carbamate and the aromatic amide (NGx03 and NGx07). NGx03 having both, an aromatic amide and additionally a trifluoromethyl substituted phenyl, displays the highest activity. The data density obtained by the limited SAR by inventory did not allow the building of a high-resolution pharmacophore template. However, tolerated substitutions of the aromatic ring system demonstrated that NGx04 is amenable to derivatization and comprises an attractive starting point for a medicinal chemistry program. In addition, NGx03 was also found to inhibit growth of a FLU-resistant *C*. *neoformans* strain (S3 Fig) and furthermore the effect was found to be cidal S6 Fig), underlining the potential of ergolines to fight *C*. *neoformans* infections.

Some of the compounds presented here were also tested against *C*. *albicans* (ATCC 24433), *A*. *fumigatus* (ATCC MYA-3627), R. oryzae (ATCC MYA-4621) and *F*. *solani* (ATCC MYA-3636), but found to be inactive (S8 Fig). Since the fungal Sec14 proteins are evolutionary conserved, targeting Sec14p seems only to be efficacious in *C*. *neoformans*. Sec14p-dependent virulence has only been described for *C*. *neoformans* [11] but not for other fungal pathogen. It remains to be seen if in the future intervening at different nodes of the cellular lipid transfer pathway might yield novel anti-fungal targets.

From a chemical perspective one might suspect some NGx side groups to be potentially reactive. We thus tested the cryptococcal active compounds in a rat liver microsome-based assay for metabolic stability but could not detect significant degradation (S7 Fig). In addition, potent activity in rich medium (containing crude cell extracts) and the absence of any cell-wall/plasma membrane-stress indicative hits in HIP and HOP [26, 58], support the notion that this scaffold is stable and selectively targeting Sec14p.

Invasion of the central nervous system by *C*. *neoformans* is a prerequisite for fungal meningitis. We thus performed artificial membrane permeability assays in order to assess the potential of the cryptococcal active compounds to penetrate the blood brain barrier (S7 Fig). All tested compounds, with the exception of NGx13, showed high permeability indicating a potential exposure of the CNS. High permeability of ergolines is expected, as members of the ergoline class are used as psychedelic narcotics since their discovery.

Although hallucinogenic and toxic effects of ergot alkaloids are known since 600 BC [59], a first representative was isolated only in 1875 [60]. Today, over 80 ergot alkaloids from fungi and plants have been described [61] and a number non-hallucinogenic ergolines, or their derivatives, are used in the clinic to treat Parkinson’s disease, acromegaly, hyperprolactinemia, restless-legs syndrome, hypotony, migraine and other diseases (summarized by [61]). Interestingly, the effects of this substance class are exclusively transmitted via α-adreno-, serotonin- or dopamine receptors in mammals [62]. Identification of NGx04 as Sec14p inhibitor not only provides the first report of ergolines as non-GPCR modulators, but also the first activity of ergolines on lower eukaryotes.

## Materials and Methods

### Compound handling

Compounds were stored as powders until use. Prior to testing compounds were dissolved as 10 mM stocks into 90% DMSO and kept at 4°C for a maximum of 6 months. Purity and integrity was analyzed by LC-MS/MS and UV. Solutions only passed QC if corresponding mass could be identified and the main component concentration was above 85%.

### *S*. *cerevisiae* strains

The heterozygous and homozygous deletion strain collections were acquired (OpenBiosystems YSC1055 and YSC1056) and pools generated as described previously [63]. The functional variomics collection was obtained from Dr. X. Pan (Baylor College of Medicine, Houston, TX 77030, USA).

### *C*. *neoformans* strains

*C*. *neoformans* strains ATCC 36556, a lung isolate, and ATCC 90113, a cerebrospinal fluid isolate, were purchased from ATCC, Manassas, VA (USA). BPY22 was obtained from [45].

### Haploinsufficiency profiling (HIP) and homozygous profiling (HOP)

Genomic profiling and microarray analysis were performed as described previously [26].

### Functional variomics screen

The functional variomics screen was performed as described previously [34] with the difference that plasmids of resistant clones were analyzed by Sanger sequencing and not chip hybridization: Clones were picked and grown over night in 1 ml of YPD at 30°C in a 96 deep well block. Cells were then subsequently washed with distilled water and subjected to a zymolyase treatment prior to purification of plasmid DNA by Wizard SV DNA purification system (Promega)

### Phosphatidylinositol (PI) and phosphatidylcholine (PC) competition

*S*. *cerevisiae* BY4741 (Open Biosystems) was grown in YPD medium in the absence or presence of additional phosphatidylinositol or phosphatidylcholine (200 μM each, Sigma-Aldrich 79401 or P3556, respectively). OD600 values of exponentially growing yeast cultures were recorded with a robotic system. Twelve-point serial dilutions were assayed in 96-well plates with a reaction volume of 150 μl.

### Crystal structure docking

The *S*. *cerevisiae* protein structure (PDB ID 1AUA) has been prepared using the default settings of the ProteinPreparation Wizard within the Maestro Schrödinger Suite (www.schrodinger.com). Ligand coordinates have been generated with Corina [64]. This conformation has been used as a starting point for the docking. The docking procedure was carried out using Glide SP within the Maestro Schrödinger Suite. After docking the complex protein-ligand has been minimized using MacroModel within the Maestro Schrödinger Suite, allowing the ligand to be flexible and keeping the protein rigid.

### Mammalian cell growth inhibition

Profiling across the Cancer Cell Line Encyclopedia was performed as described previously [65]. MTS toxicity was determined using a CellTiter 96 AQ_ueous_ One Solution Cell Proliferation Assay kit from Peomega.

### *C*. *neoformans* growth inhibition

Antifungal susceptibility testing was conducted according to the Clinical and Laboratory Standards Institute (CLSI) guidelines for broth microdilution assay M27-A3. Testing was performed in RPMI-1640 (HyClone) with 2.05 mM glutamine, phenol red, without bicarbonate and buffered with 0.165 mol/l 3-(N-morpholino) propanesulfonic acid (MOPS). The medium was adjusted to pH 7.0 and sterilized with a 0.2 μm filter. Two endpoints were recorded: MIC-0 (complete inhibition with no visible fungal growth) and MIC-2 (prominent inhibition corresponding to >50% growth reduction).

### Fungicidal vs fungistatic drug testing

To test the fungistatic or fungicidal effect of the ergolines, the format of the CLSI susceptibility assay was followed. Briefly, *C*. *neoformans* strains were grown overnight in YEPD medium at 30°C under constant agitation. Cells were then diluted to about 10^3^ cells/ml in RPMI medium as starting inoculum and 200 μl of this inoculum was pipetted in 96- well flat bottom microtiter plate. The starting inoculum was subjected to CFU counts in YEPD agar by plating serial dilutions and 48 hrs. incubation. Drugs were diluted to a final concentration of 16μg/ml and MIC-0 values determined by the CLSI protocol. After an incubation of 24 hrs. at 35°C, cells were diluted in RPMI and plated onto YEPD agar for CFU counting. A fungicidal effect was defined as decreased CFUs in drug-exposed cultures as compared to CFUs in the starting inoculum.

### Rat liver microsome and artificial membrane permeability assay

Metabolic stability was determined as described previously [66] on a fully automated platform, the artificial membrane permeability assays was performed as described previously [67]

## Supporting Information

S1 Fig Protein comparison 1. **Alignment of CnSec14-1p, ScSec14p and HsSec14-2p.** The CLUSTAL 2.1 alignment of *C*. *neoformans*, *S*. *cerevisiae* and *H*. *sapiens* Sec14 proteins is shown and the NGx04 resistance conferring amino acids highlighted in red.
(EPS)

S2 Fig Protein comparison 2. **Alignment of ScSec14p, CnSec14-1p and CnSec14-2p.**The CLUSTAL 2.1 alignment of *S*. *cerevisiae* Sec14p and *C*. *neoformans* CnSec14-1p and CnSec14-2p is shown. Red boxes represent conserved amino acids involved in NGx04 binding in the three proteins, blue boxes represent non-conserved amino acids.
(EPS)

S3 Fig Anti-fungal resistance testing. **Inhibition of fluconazole resistant *C*. *neoformans*.** NGx04 and close analogues were tested against the FLU-resistant *C*. *neoformans* BPY22 [45] in a 48 h proliferation assay and MIC-2 (50% inhibition) and MIC-0 (no visible fungal growth) determined. Values are given in μM as mean of tree replicates. Fluconazole was included as control to show hyper-resistance of BPY22.
(EPS)

S4 Fig Resistance mapping. **Functional variomics screen for NGx04-resistant mutants.** Statistics of the primary screen (genome-wide pool) and the secondary screen (SEC14 pool) are displayed. 1.5 OD_600_ of the corresponding pools were plated on a 15 cm SD agar dish containing 150 μM NGx04, resistant clones isolated and analyzed by sequencing of the expressed ORF. Numbers in parenthesis display the frequency of the isolated ORFs.
(EPS)

S5 Fig Evolutionary divergence. **Complementation of yeast SEC14.** A heterozygous *SEC14/sec14 S*. *cerevisiae* strain was transformed with the human SEC14 homologue (XP_011510441.1) under the control of the ADH1 promotor. Transformants were isolated, subjected to sporulation, tetrads dissected and analysed for viability. 18 tetrads are shown (horizontal). Only two viable spores per tetrad were found, indicating no genetic complementation.
(EPS)

S6 Fig Effectiveness of growth inhibition. **Fungicidal vs fungistatic effects of ergolines.** Ergolines were tested at 16 μg/ml final concentration for 24 hrs. against FLU-sensitive (DSY4982) and FLU-resistant (BPY22 and BPY17) *C*. *neoformans* isolates [45] for fungicidal activity and Colony Forming Units in presence (NGxXX) and in absence (inoculum) of compound are displayed as mean of three replicates. Detection limit of the assay was 10^2^ cells/ml. This value was attributed to samples in which this detection limit was reached.
(EPS)

S7 Fig Physicochemical properties of ergolines. **Rat liver microsome and artificial membrane permeability assay.** NGx04 and close analogues were tested for metabolic stability (CYP MetCL) and artificial membrane permeability (logPAMPA) in duplicates. Value pairs are color coded and binning is indicated.
(EPS)

S8 Fig Anti-fungal testing. **Fungal pathogen assay.** NGx04 and close analogues were tested against *C*. *albicans* (ATCC 24433), *A*. *fumigatus* (ATCC MYA-3627), *R*. *oryzae* (ATCC MYA-4621) and *F*. *solani* (ATCC MYA-3636) in a 72 h proliferation assay and MIC-2 (50% inhibition) and MIC-0 (no visible fungal growth) determined. Values are given in μM as mean of tree replicates. Amphotericin B was included as positive control.
(EPS)
